# Utility of admission biomarkers in predicting severe outcomes and triage in acute febrile illness: A cohort study

**DOI:** 10.1177/03000605251375552

**Published:** 2025-10-15

**Authors:** Thejesh Srinivas, Nitin Gupta, Gagana Hanumaiah, Shwethapriya R, Prithvishree Ravindra, Kavitha Saravu, Ravindra Maradi, Souvik Chaudhuri

**Affiliations:** Kasturba Medical College, Manipal Academy of Higher Education, India

**Keywords:** Acute febrile illness, admission biomarkers, triage, invasive mechanical ventilation, multiorgan dysfunction

## Abstract

**Objectives:**

To determine the role of biomarkers in acute febrile illness patients at admission in predicting moderate-to-severe multiorgan dysfunction at 24 h of hospitalization and the need for invasive mechanical ventilation at 48 h of hospitalization.

**Methods:**

This prospective cohort study was conducted among 100 acute febrile illness patients brought to the emergency department. Biochemical and clinical parameters at hospital admission were recorded. The highest Sequential Organ Failure Assessment score was calculated at 24 h of hospitalization. The need for invasive mechanical ventilation at 48 h of hospitalization was evaluated.

**Results:**

Of the 95 acute febrile illness patients, 60 (63.15%) had moderate-to-severe multiorgan dysfunction. Multivariable logistic regression showed that admission aspartate aminotransferase level ≥89 U/L (P < 0.001; area under the curve, 0.752), C-reactive protein level ≥161 mg/dL (P < 0.001; area under the curve, 0.751), and urea level ≥74 mg/dL (P < 0.001; area under the curve, 0.855) were independent predictors of moderate-to-severe multiorgan dysfunction at 24 h. Serum interleukin-6 level ≥84.48 pg/mL (P = 0.002; area under the curve, 0.728) on admission was an independent predictor of the need for invasive mechanical ventilation.

**Conclusions:**

Urea, aspartate aminotransferase, and C-reactive protein levels on admission may independently predict moderate-to-severe multiorgan dysfunction in acute febrile illness patients at 24 h of hospitalization. In addition, interleukin-6 level may be an independent predictor of the need for invasive mechanical ventilation at 48 h of hospitalization.

## Introduction

Approximately 35% of patients with acute febrile illness (AFI) may progress to multiorgan dysfunction (MOD), with the failure of two or more organs.^1,2^ Early detection of MOD risk helps clinicians administer optimal treatment when the organ dysfunction is still at a reversible stage.^3,4^ With the use of algorithms, a “lead time” of approximately 22 h for MOD helps clinicians identify at-risk patients who may not otherwise be recognized at an early stage.^4^ Although clinical scores such as the quick Sequential Organ Failure Assessment (SOFA) score (respiratory rate ≥22/min, systolic blood pressure ≤100 mmHg) and Glasgow coma scale score (<15) can identify patients with a higher risk of deterioration, they have limitations in cases of severe infections and inflammatory conditions.^3^ In such scenarios, biomarkers often help in the early prediction of MOD in conjunction with clinical parameters.^3^ The SOFA score calculation for predicting the severity of MOD entails the selection of the worst individual organ dysfunction parameters within a 24-h period.^5^

In AFI patients, if the admission biomarkers can predict moderate-to-severe MOD at 24 h, such markers should be focused on by clinicians. This may help in predicting the need for intense monitoring.

We have previously published one aspect of this research using the same patient cohort, which focused on the utility of point-of-care ultrasound (to assess pulmonary, cardiac, and renal parameters) and arterial blood gas estimation in guiding disposition decisions in the emergency department (ED), including the need for continuous vital monitoring and admission to either the intensive care unit (ICU) or high-dependency unit.^6^ In contrast, the present study addresses an entirely different, prospectively planned research question, specifically examining the role of admission biomarkers in predicting moderate-to-severe organ dysfunction (SOFA score: 8–24) at 24 h and the need for invasive mechanical ventilation within 48 h in AFI patients.

### Aim

To determine the utility of biomarkers at admission in AFI patients for early prediction of moderate-to-severe organ dysfunction.

### Primary objective

To determine the value of biomarkers at admission in predicting the development of moderate-to-severe MOD within 24 h of hospitalization in patients with AFI.

### Secondary objective

To determine the value of biomarkers at admission in predicting the requirement for invasive mechanical ventilation (IMV) within 48 h of hospitalization in patients with AFI.

### Primary outcome

The primary outcome was progression toward moderate-to-severe organ dysfunction (SOFA score: 8–24) at 24 h of hospitalization among hospitalized AFI patients.

### Secondary outcome

The secondary outcome was the need for IMV within 48 h of hospitalization among hospitalized AFI patients.

## Methodology

This was a single-center prospective cohort study among 100 AFI patients. The study was conducted from August 2022 to December 2022 at a 2032-bedded tertiary healthcare set-up after obtaining approval from Kasturba Medical College and Kasturba Hospital Institutional Ethics Committee located at Dean Office block, Kasturba Medical college, Manipal campus, Manipal Academy of Higher Education, Manipal, on 3 July 2022 with approval number: IEC 56/2022; the study was registered in the Clinical Trials Registry India, CTRI/2022/07/044318, on 26 July 2022. The reporting of this study conforms to the Strengthening the Reporting of Observational Studies in Epidemiology (STROBE) guidelines.^7^ (Supplementary material 1). We have conducted this study in accordance with the Helsinki Declaration of 1975, as revised in 2024.

The sample size was calculated based on an expected sensitivity of the biomarker in predicting MOD at 24 h of hospitalization (estimated to be 90%). Assuming a 95% confidence interval (CI) (Z = 1.96), a 5% α-error, and an allowable margin of error of 6%, the required sample size was determined using the following formula:^8^

$$n=\frac{Z^{2}\text{×}\;p\;\text{×}\;(1-p)}{E^{2}}$$

In the above formula, n is the required sample size, Z is the standard normal deviate for the given confidence level (1.96 for 95% CI), p is the expected sensitivity (0.90), and E is the allowable margin of error (0.06). This method ensures an adequate sample size to provide reliable estimates of the biomarker’s diagnostic performance. The calculated sample size using this method was 96, which was approximated to 100. Therefore, we included 100 patients in this study.

AFI was defined as a fever above 101°C lasting for less than 15 days, without a localized source of infection, accompanied with either thrombocytopenia, leukopenia, or leukocytosis.^9^ For the purposes of this study, moderate-to-severe MOD was defined by a SOFA score ranging from 8 to 24 at 24 h of hospitalization. Similarly, the requirement for IMV was defined as the need for intubation and mechanical ventilation within 48 h of hospital admission. These operational definitions were used to ensure consistency in patient classification and facilitate accurate comparisons within the study cohort.

The study inclusion criteria included patients aged over 18 years, those of any sex, and those who were recruited within 24 h of hospitalization for determining the levels of inflammatory biomarkers, specifically interleukin-6 (IL-6), neutrophil gelatinase–associated lipocalin (NGAL), and inducible nitric oxide synthase (iNOS). The patients were included in the study through consecutive sampling.

**Figure 1.**
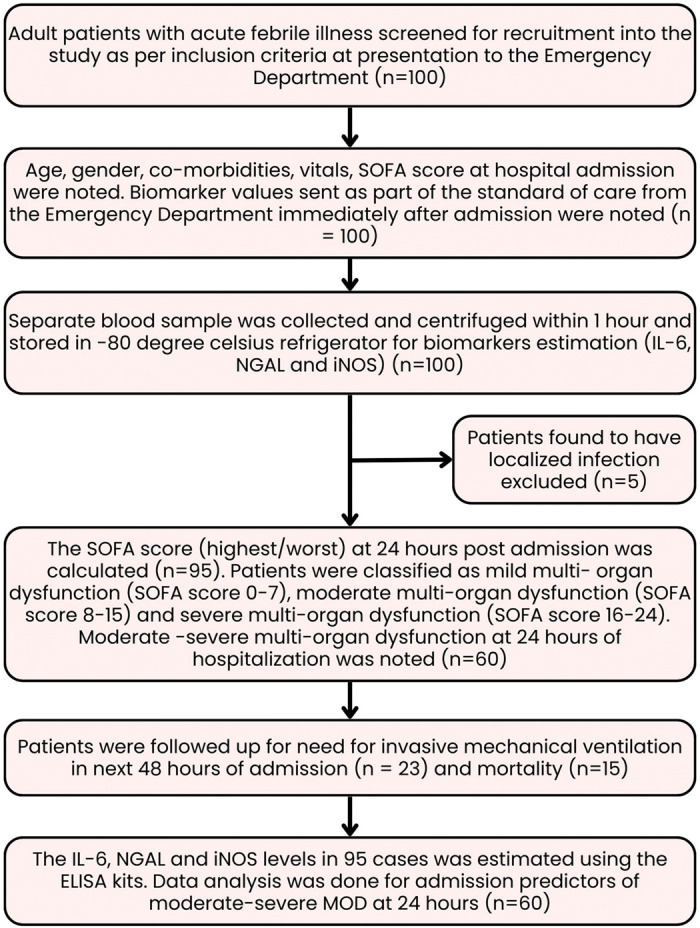
Flowchart illustrating the process of acute febrile illness patients’ enrollment into the study.

The exclusion criteria included patients with a localized source of infection, those with prior hospital admission for >48 h within the last 15 days, and those with chronic kidney disease with a glomerular filtration rate <60 mL/min. Additionally, patients with ischemic heart disease, pulmonary artery hypertension, and autonomic dysfunction as well as those with terminal illness who were unlikely to survive for at least 48 h were excluded. Patients were also excluded if biochemical investigations, including blood tests, renal parameters, and liver function tests, were unavailable immediately after admission to the ED. Additionally, patients receiving corticosteroid therapy were excluded due to its potential immunosuppressive effects on biomarker elevation. At the time of ED admission, patients were enrolled in the study only after obtaining written informed consent from legally recognized patient representatives. We have deidentified all details of the patients included in our study. The Consolidated Standards of Reporting Trials (CONSORT) diagram of the study is depicted in Figure 1.

Demographic and clinical data, including age, sex, Charlson comorbidity index scores, and SOFA scores were recorded. Baseline laboratory parameters such as white blood cell (WBC) and platelet counts as well as aspartate aminotransferase (AST), C-reactive protein (CRP), procalcitonin (PCT), urea, creatinine, sodium, potassium, total bilirubin, hemoglobin levels were documented at the time of admission. Blood samples were collected, centrifuged at 5000 r/min, and then stored at −80°C for subsequent biomarker analysis. Biomarkers including IL-6, NGAL, and iNOS were quantified using enzyme-linked immunosorbent assay (ELISA) kits. The standardization process of the ELISA kits enabled the determination of IL-6 levels in 87 patients, NGAL levels in 85 patients, and iNOS levels in 86 patients. The standard curve ranges and sensitivities for the assays were as follows: IL-6 (6.25–200 pg/mL, sensitivity: 2 pg/mL), NGAL (5–600 ng/mL, sensitivity: 2.01 ng/mL), and iNOS (0.5–200 U/L, sensitivity: 0.23 U/L). The highest SOFA score at 24 h of hospitalization was calculated, and patients were classified as having mild (SOFA score: 0–7), moderate (SOFA score: 8–15), and severe (SOFA score: 16–24) MOD.^10^ The diagnosis of AFI was noted, along with the need for IMV and mortality.

### Statistical analyses

The data were analyzed using Statistical Package for the Social Sciences (SPSS) version 29.0 (IBM SPSS Statistics for Windows, version 6.0, Armonk, NY: IBM). For variables with a parametric distribution, the mean ± SD values were reported, while for nonparametric variables, the median and interquartile range (IQR) values were calculated. The independent Student t-test was employed to compare the mean values between the two groups, and the Mann–Whitney U test was used to compare median values.

To identify the predictors of outcomes, variables with a P-value ≤0.05 in the independent Student t-test or Mann–Whitney U test were included in a univariate analysis, where odds ratios (OR) were calculated. Variables with a P-value ≤0.1 in the univariate analysis were included in multivariable logistic regression to determine independent predictors and their adjusted ORs (AORs). Variables that remained significant after multivariable logistic regression (P ≤ 0.05) were identified as independent predictors of specific outcomes.

These significant variables were subsequently used to construct receiver operating characteristic (ROC) curves. The area under the curve (AUC), P-value, cutoff value, sensitivity, specificity, and 95% CI were determined for variables to evaluate their performance in predicting the outcomes of interest.

## Results

Of the 100 AFI patients, 5 were excluded because they had localized infections, and a final cohort of 95 patients was analyzed. The mean age of the patients was 48.79 ± 15.18 years, and 71.6% of them were men. A confirmed diagnosis of AFI was established for 64 patients (67.36%), while the remaining 31 patients had AFI without a specific diagnosis (Table 1). The highest SOFA score at 24 h was 9.1 ± 4.17. Moderate-to-severe MOD was observed in 60 patients (63.15%) at 24 h, and 23 patients (24.21%) required IMV during hospitalization (Table 1). The initial admission biomarkers of AFI patients with mean ± SD or median (IQR) are displayed in Table 2.

**Table 1.** Demographics and characteristics of AFI patients (N = 95).

Variables	Mean ± SD/Median (IQR)/ Number (%)
Age (years)	48.79 ± 15.18
Confirmed diagnosis of AFI, n (%)	64 (67.36%)
AFI with nonspecific diagnosis, n (%)	31 (32.63%)
Highest SOFA score at 24 h of hospitalization	9.1 ± 4.17
Mild organ dysfunction (SOFA score: 0–7), n (%)	35 (36.84%)
Moderate organ dysfunction (SOFA score: 8–15), n (%)	52 (54.73%)
Severe organ dysfunction (SOFA score: 16–24), n (%)	8 (8.42%)
Moderate-to-severe organ dysfunction (SOFA score: 8–24), n (%)	60 (63.15%)
IMV needed, n (%)	23 (24.21%)
NIV needed, n (%)	22 (23.15%)
Mortality, n (%)	15 (15.7%)
Charlson comorbidity index score	1 (0–2)
qSOFA score at admission to ED	2 (1–2)
High qSOFA ≥ 2 in AFI patients at ED admission, n (%)	50 (52.6%)
Heart rate (beats/min)	100 ± 18.8
SBP (mmHg)	112.20 ± 20.75
SpO_2_ (%)	94 ± 5
Leptospirosis, n (%)	46 (48.4%)
Scrub typhus, n (%)	11(11.6 %)
Dengue, n (%)	7 (7.4%)

IQR: interquartile range; AFI: acute febrile illness; SOFA: Sequential Organ Failure Assessment; IMV: invasive mechanical ventilation; NIV: noninvasive ventilation; qSOFA: quick Sequential Organ Failure Assessment; ED: emergency department; SBP: systolic blood pressure; SpO_2_: oxygen saturation.

**Table 2.** Admission biomarkers in AFI patients in the emergency department (N = 95).

Variables	n	Mean ± SD/Median (IQR)
IL-6 (pg/mL)	87	70.43 ± 30.67
NGAL (ng/mL)	85	163.93 ± 36.19
iNOS (U/L)	86	55.33 ± 13.99
Hemoglobin (g/dL)	95	11.91 ± 2.48
Sodium (mEq/L)	95	132.32 ± 6.74
Potassium (mEq/L)	95	4.34 ± 0.87
Urea (mg/dL)	95	67 (41–119)
Creatinine (mg/dL)	95	2.19 (1.21–3.97)
Platelet count (10^3^/µL)	95	41.5 (19–99)
WBC count (10^3^/µL)	95	9.2 (6.3–14.30)
Total bilirubin (mg/dL)	95	3.1 (0.93–6.72)
AST (IU/L)	95	99 (52.50–176.75)
CRP (mg/dL)	91	186 (108–286)
PCT (ng/mL)	84	7.57 (1.45–38.9)

AFI: acute febrile illness; IQR: interquartile range; IL-6: interleukin-6; NGAL: neutrophil gelatinase–associated lipocalin; iNOS: inducible nitric oxide synthase; WBC: white blood cell; AST: aspartate aminotransferase; CRP: C-reactive protein; PCT: procalcitonin.

To predict MOD at 24 h using admission biomarkers, parameters such as creatinine, total bilirubin, and platelet count, which are components of the SOFA score, were excluded to avoid bias. A comparison between patients with mild MOD and those with moderate-to-severe MOD revealed significant differences in admission values of AST (P = 0.001), WBC (P = 0.023), CRP (P < 0.001), PCT (P < 0.001), and urea (P < 0.001) (Supplementary Material 2). Univariate and multivariable logistic regression analyses identified AST, CRP, and urea at admission as independent predictors of moderate-to-severe MOD in AFI patients at 24 h of hospitalization (Table 3).

**Table 3.** Univariate and multivariable logistic regression analyses of the initial admission biomarkers to predict moderate-to-severe MOD (n = 60) at 24 h in AFI patients.

Univariate analysis				Multivariable logistic regression		
Biochemical parameters	P	OR	95% CI	P	AOR	95% CI
AST (IU/L)	**0.030**	1.00	1.000–1.008	**0.006**	1.00	1.002–1.010
WBC count (10^3^/µL)	**0.048**	1.00	1.000–1.000	0.703	1.00	1.000–1.000
CRP (mg/dL)	**<0.001**	1.01	1.005–1.015	**0.006**	1.01	1.003–1.017
PCT (ng/mL)	0.402	1.00	0.995–1.013			
Urea (mg/dL)	**<0.001**	1.03	1.017–1.048	**0.003**	1.03	1.010–1.059

AFI: acute febrile illness; WBC: white blood cell; AST: aspartate aminotransferase; CRP: C-reactive protein; PCT: procalcitonin; MOD: multiorgan dysfunction; CI: confidence interval; OR: odds ratio; AOR: adjusted odds ratio.

Variables with a P-value ≤0.1 in univariate analysis were included in multivariable logistic regression.

P ≤ 0.05 was considered to indicate statistical significance (values marked in bold).

**Figure 2.**
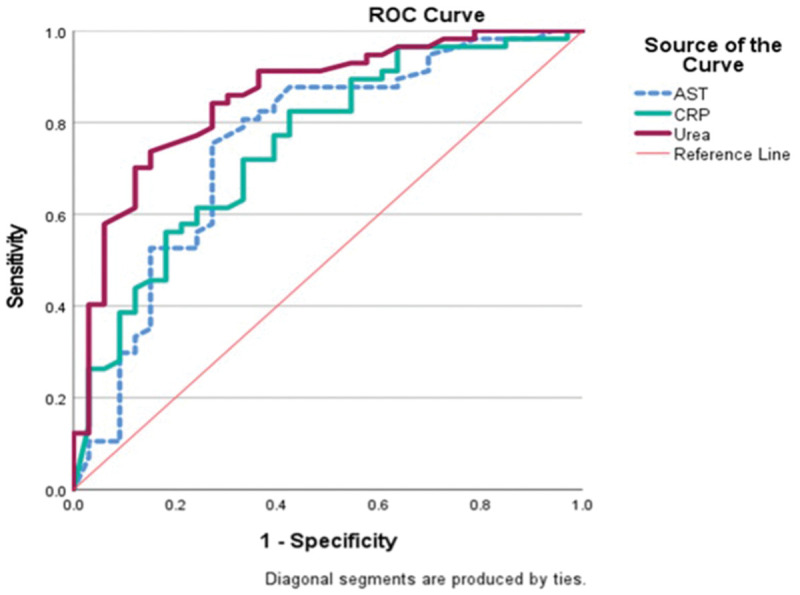
The ROC curve of the independent predictors (AST, CRP, and urea) in AFI patients with moderate-to-severe MOD at 24 h of hospitalization. AFI: acute febrile illness; AST: aspartate aminotransferase; CRP: C-reactive protein; MOD: multiorgan dysfunction; ROC: receiver operating characteristic. P ≤ 0.05 was considered to indicate statistical significance (values marked in bold). Urea: AUC, 0.855; P < 0.001; 95% CI: 0.774–0.937; cutoff, ≥74 mg/dL; sensitivity, 70%; specificity, 88%. AST: AUC, 0.752; P < 0.001; 95% CI: 0.641–0.863; cutoff, ≥89 U/L; sensitivity, 70%; specificity, 73%. CRP: AUC, 0.751; P < 0.001; 95% CI: 0.646–0.856; cutoff, ≥161 mg/dL; sensitivity, 72%; specificity, 67%.

A urea cutoff level of ≥74 mg/dL demonstrated an AUC of 0.855 (P < 0.001, 95% CI: 0.774–0.937), with 70% sensitivity and 88% specificity for predicting moderate-to-severe MOD (Figure 2). Similarly, an AST cutoff level of ≥89 IU/L showed an AUC of 0.752 (P < 0.001, 95% CI: 0.641–0.863), with 70% sensitivity and 73% specificity. For CRP, a cutoff level of ≥161 mg/dL yielded an AUC of 0.751 (P < 0.001, 95% CI: 0.646–0.856), with 72% sensitivity and 67% specificity (Figure 2).

**Figure 3.**
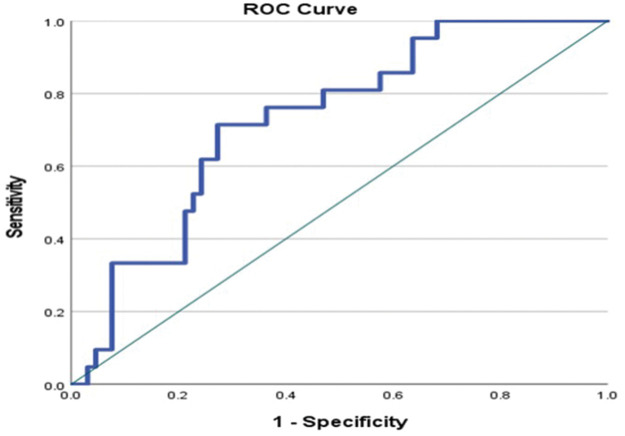
The ROC curve of IL-6 levels at admission to predict the need for IMV at 48 h of hospitalization in AFI patients. AFI: acute febrile illness; IMV: invasive mechanical ventilation; IL-6: interleukin-6; ROC: receiver operating characteristic. P ≤ 0.05 was considered to indicate statistical significance (values marked in bold). AUC, 0.728; P = 0.002; 95% CI: 0.614–0.842; cutoff, ≥84.48 pg/mL; sensitivity, 71%; specificity, 72.7%.

Comparison of AFI patients requiring IMV within 48 h of hospitalization versus AFI patients not requiring IMV within 48 h of admission showed that there was a significant difference between the admission values of AST (P = 0.016), WBC (P = 0.019), urea (P = 0.006), creatinine (P = 0.004), potassium (P = 0.005), and IL-6 (P = 0.003), as shown in Supplementary Material 3. Univariate and multivariable logistic regression analyses showed that IL-6 was an independent predictor of the need for IMV within 48 h of hospitalization in AFI patients (P = 0.020) (Table 4). The ROC curve of IL-6 to predict IMV requirement showed the following results: AUC, 0.728; P = 0.002; 95% CI, 0.614–0.842; cutoff level, ≥84.48 pg/mL; sensitivity, 71%, and specificity, 72.7% (Figure 3).

**Table 4.** Univariate and multivariable logistic regression analyses of the initial admission biomarkers to predict the need for IMV within the next 48 h after hospitalization.

Univariate analysis				Multivariable logistic regression		
Biochemical parameters	P	OR	95% CI	P	AOR	95% CI
AST	**0.09**	1.00	1.000–1.005	0.324	1.00	0.998–1.005
WBC count	**0.087**	1.00	1.000–1.000	0.754	1.00	1.000–1.000
Potassium	**0.009**	2.14	1.212–3.788	0.221	1.57	0.761–3.260
IL-6	**0.006**	1.03	1.008–1.051	**0.020**	**1.02**	**1.004–1.050**
Urea	**0.029**	1.00	1.001–1.017	0.861	1.00	0.985–1.018
Creatinine	**0.031**	1.26	1.022–1.575	0.759	1.07	0.692–1.655

IMV: invasive mechanical ventilation; IL-6: interleukin-6; WBC: white blood cell; AST: aspartate aminotransferase; CI: confidence interval; OR: odds ratio; AOR: adjusted odds ratio.

Variables with a P-value ≤0.1 in univariate analysis were included in multivariable logistic regression.

P ≤ 0.05 was considered to indicate statistical significance (values marked in bold).

**Figure 4.**
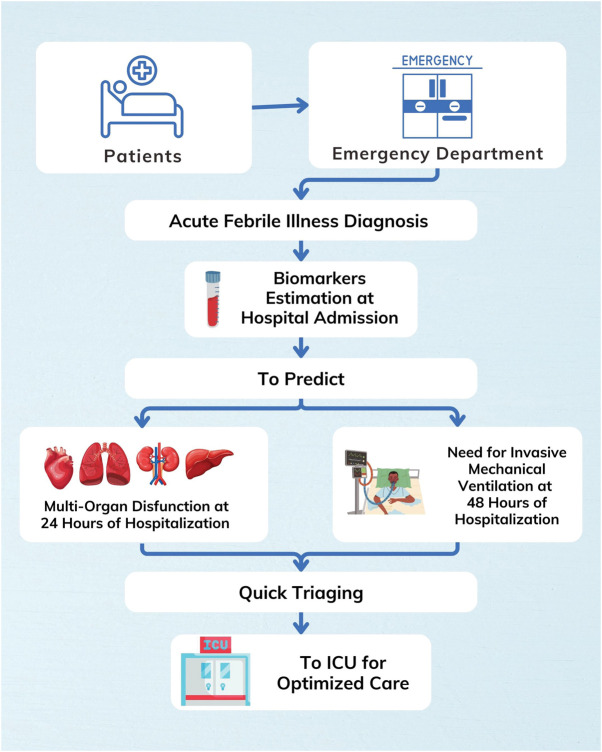
Graphical summary of the study.

The cutoff levels, sensitivity, and specificity of the admission biomarkers in predicting moderate-to-severe organ dysfunction and the need for IMV at 48 h of hospitalization are depicted in Table 5. Figure 4 provides a graphical summary of the study.

**Table 5.** Depiction of the independent predictors of the need for IMV at 48 h and moderate-to-severe MOD at 24 h of hospitalization in AFI patients.

Independent biomarkers at admission for predicting need for IMV at 48 h of hospitalization						
Variable	AUC	P	95% CI	Cutoff	Sensitivity	Specificity
IL-6	0.728	**0.002**	0.614–0.842	≥84.48 pg/mL	71%	72.7%
Independent biomarkers at admission for predicting moderate-to-severe organ dysfunction at 24 h of hospitalization						
Urea	0.855	**<0.001**	0.774–0.937	≥74 mg/dL	70%	88%
AST	0.752	**<0.001**	0.641–0.863	≥89 IU/L	70%	73%
CRP	0.751	**<0.001**	0.646–0.856	≥161 mg/dL	72%	67%

IMV: invasive mechanical ventilation; MOD: multiorgan dysfunction; AFI: acute febrile illness; IL-6: interleukin-6; AST: aspartate aminotransferase; CRP: C-reactive protein; AUC: area under the curve; CI: confidence interval.

P ≤ 0.05 was considered to indicate statistical significance (values marked in bold) based on multivariable logistic regression analysis.

## Discussion

Clinicians must identify the risk of MOD as early as possible, even if it is not initially evident at the time of ED admission.^4^ Although clinical parameters can help predict early warning signs, they are not universally reliable in all situations, and incorporating biomarkers may offer additional value.^3^ Moreover, the level of inflammatory biomarkers often increase before the SOFA score rises and MOD develops, providing clinicians with a critical window to intervene effectively before MOD progresses to an irreversible stage.^3,11^

With this background, we aimed to identify independent biomarkers at the time of admission that could predict the development of moderate-to-severe MOD within 24 h. In MOD caused by AFI, organ dysfunction typically affects the pulmonary, renal, coagulation, hepatobiliary, and neurological systems.^1^ Consequently, the SOFA score serves as an optimal tool to assess the extent of organ dysfunction in AFI patients because it encompasses all these systems.^5^ Biomarkers associated with these organ dysfunctions were assessed upon ED admission to offer clinicians an early warning of MOD. None of the biomarkers assessed are part of the SOFA score, which is used for determining the severity of organ dysfunction.

Serum urea level at admission was an independent predictor of MOD at 24 h in our study. This could be because in AFI, especially leptospirosis (which affected approximately 50% of our AFI patients), oliguria and acute kidney injury (AKI) occur mainly due to dehydration.^12^ Thus, AKI is often associated with a rapid rise in the urea level, along with a rise in bilirubin level.^13^ AKI in AFI, which is caused by a hypercatabolic process, with raised blood urea nitrogen and creatinine levels, may progress from the nonoliguric to oliguric phase if adequate hydration and hemodynamic goals are not addressed on ED admission.^14^

AST was identified as an independent biomarker for predicting MOD at 24 h. Our findings aligned with those reported in the literature, which revealed that elevated AST levels during the immune stage of leptospirosis (days 4–30 of illness) is an indicator of disease severity.^15^ Additionally, a high AST/alanine aminotransferase (ALT) ratio (>3) in leptospirosis patients is associated with poor outcomes.^13^ Similarly, in scrub typhus, elevated AST levels have been linked to severe disease.^16^

The admission CRP level was also an independent predictor of MOD at 24 h. CRP is primarily secreted by hepatocytes in response to an inflammatory state, explaining its role as a biomarker of severe organ dysfunction in AFIs such as leptospirosis and scrub typhus.^17^ CRP levels start rising by 6 h of inflammation initiation and peak at 48–50 h; CRP levels have been shown to predict severity in various infectious and noninfectious conditions.^17^ A CRP level >50 mg/dL has been reported to predict AFI due to leptospirosis, and early antibiotic therapy is recommended in such cases.^18^ Patients with AFI also present with a worsening neurological response, an independent predictor of disease severity and MOD in AFI.^1^ CRP has also been proven as an outcome predictor in other neurological disorders such as subarachnoid hemorrhage and ischemic stroke; thus, it may serve as a predictor of not only the severity of inflammation and sepsis in AFI but also the worsening neurological function in AFI.^19,20^

IL-6 was identified as an independent predictor of the need for IMV in AFI patients within 48 h of admission. It plays a crucial role in various pulmonary conditions, including bronchial asthma and obstructive airway disease, where it is secreted by airway epithelial cells and macrophages.^21^ Additionally, IL-6 contributes to increased pulmonary resistance, thereby making breathing more difficult.^22^ IL-6 has also been identified as a predictor of the need for IMV in coronavirus disease 2019 (COVID-19) patients, with a cutoff value of >80 pg/mL, which is slightly lower than the threshold determined in our study (approximately 84.5 pg/mL).^23^ Studies suggest that IL-6 levels are correlated with the extent of pulmonary damage, providing a “window” for clinicians to assess lung involvement.^23,24^ Therefore, monitoring IL-6 levels could help ED clinicians in early identification of patients who may require ventilatory support during hospitalization.

Recent advances in other clinical domains underscore the expanding role of biomarkers in early risk stratification. For instance, evidence from acute cardiovascular and hypertensive settings reveals that novel serum and urinary biomarkers can detect organ injury before conventional clinical signs emerge.^25^ In the context of acute severe hypertension, these biomarker-driven approaches are being explored to guide management and potentially refine guidelines.^25,26^ Additionally, high-sensitivity troponin T (hs-TnT), a well-established marker of myocardial injury, has demonstrated prognostic value beyond acute coronary syndromes, even in patients with severe aortic stenosis undergoing valve replacement, where elevated pre- and postprocedural levels are correlated with a longer ICU stay and higher mortality risk.^27^ These findings suggest that incorporating biomarkers such as hs-TnT and other biomarkers indicative of acute organ injury (such as those observed in hypertension) can enhance early warning systems.^25–27^ In the context of AFI, this supports the notion that admission biomarkers such as urea, CRP, AST, and IL-6 could similarly provide a critical window for intervention, even before overt organ dysfunction develops.

One of the key strengths of this study is its focus on patients with AFI, which is a common cause of ED admission, especially during the monsoon season in tropical and subtropical regions. AFI presents with a wide spectrum of clinical manifestations, ranging from simple febrile illness to severe, life-threatening MOD. Our study highlights the predictive value of admission biomarkers such as urea, CRP, and AST in reliably identifying patients at risk of developing moderate-to-severe MOD within 24 h of hospitalization. Furthermore, through logistic regression analysis, we determined specific biomarker cutoff values, providing clinicians with actionable thresholds to enhance early risk stratification. These findings emphasize the importance of heightened clinical vigilance in AFI patients with elevated biomarker levels, potentially aiding in timely interventions to prevent irreversible MOD.

The small sample size of the current study was a primary constraint, influenced by limited financial resources for measuring IL-6, NGAL, and iNOS levels. Additionally, as this was a single-center study, the findings may not be broadly generalizable. Another limitation was the lack of biomarker trend analysis from admission to 24 h of hospitalization, which could have provided deeper insights into disease progression. Furthermore, despite categorizing patients as those with AFI, a definitive diagnosis could not be established in 31 cases (32.6%), consistent with previous studies reporting undiagnosed AFI rates of 35%–80%.^1^ Almost all patients had some degree of MOD at admission, limiting our ability to compare biomarker levels among AFI patients without MOD to determine whether elevations in biomarkers predicted MOD development or reflected existing MOD. A further limitation was that comprehensive subgroup analyses across all AFI etiologies could not be performed due to sample size constraints. Although leptospirosis and undiagnosed AFI constituted the majority of cases (48.4% and 32.6%, respectively), allowing meaningful evaluation within these groups, the smaller numbers of scrub typhus and dengue cases precluded reliable predictive modeling. Predictors identified for leptospirosis such as urea, AST, CRP, and IL-6 were largely consistent with those in the overall AFI group, suggesting a common pattern in biomarker performance across etiologies. In cases of undiagnosed AFI, the urea level remained a strong predictor of moderate-to-severe MODS, whereas IL-6 levels did not predict the need for IMV, indicating variability depending on the presence of an infection (Supplementary Material 4).

These findings underscore AFI heterogeneity and highlight the need for larger, etiology-specific studies to validate and refine biomarker predictors of MODS and IMV. Future research should focus on larger, multicenter studies with serial biomarker measurements to better understand disease progression. Integrating point-of-care biomarker testing for IL-6, CRP, urea, and AST in the ED could enable rapid clinical decision-making, allowing timely interventions before organ dysfunction becomes irreversible. As healthcare systems evolve, particularly in resource-limited settings, incorporating such technological advancements into established clinical protocols could significantly improve patient management and reduce the mortality associated with AFI-related MOD.

## Conclusion

In AFI patients, at the time of ED admission, a serum urea level ≥74 mg/dL, AST level ≥89 IU/L, and CRP level ≥161 mg/dL may independently predict the risk of developing moderate-to-severe organ dysfunction within 24 h of hospitalization. Additionally, a serum IL-6 level ≥84.48 pg/mL at ED admission may predict the need for IMV within 48 h of hospitalization. These findings highlight the potential role of early biomarker assessment in risk stratification and timely clinical intervention for AFI patients.

## Supplemental Material

sj-pdf-1-imr-10.1177_03000605251375552 - Supplemental material for Utility of admission biomarkers in predicting severe outcomes and triage in acute febrile illness: A cohort study Supplemental material, sj-pdf-1-imr-10.1177_03000605251375552 for Utility of admission biomarkers in predicting severe outcomes and triage in acute febrile illness: A cohort study by Thejesh Srinivas, Nitin Gupta, Gagana Hanumaiah, Shwethapriya R, Prithvishree Ravindra, Kavitha Saravu, Ravindra Maradi and Souvik Chaudhuri in Journal of International Medical Research

sj-pdf-2-imr-10.1177_03000605251375552 - Supplemental material for Utility of admission biomarkers in predicting severe outcomes and triage in acute febrile illness: A cohort study Supplemental material, sj-pdf-2-imr-10.1177_03000605251375552 for Utility of admission biomarkers in predicting severe outcomes and triage in acute febrile illness: A cohort study by Thejesh Srinivas, Nitin Gupta, Gagana Hanumaiah, Shwethapriya R, Prithvishree Ravindra, Kavitha Saravu, Ravindra Maradi and Souvik Chaudhuri in Journal of International Medical Research

sj-pdf-3-imr-10.1177_03000605251375552 - Supplemental material for Utility of admission biomarkers in predicting severe outcomes and triage in acute febrile illness: A cohort study Supplemental material, sj-pdf-3-imr-10.1177_03000605251375552 for Utility of admission biomarkers in predicting severe outcomes and triage in acute febrile illness: A cohort study by Thejesh Srinivas, Nitin Gupta, Gagana Hanumaiah, Shwethapriya R, Prithvishree Ravindra, Kavitha Saravu, Ravindra Maradi and Souvik Chaudhuri in Journal of International Medical Research

## Data Availability

Anonymized data will be available from the corresponding author upon reasonable request.
