# Supplementary material for: Utility of admission biomarkers in predicting severe outcomes and triage in acute febrile illness: A cohort study
Source: J Int Med Res. 2025 Oct 15;53(10):03000605251375552. doi: 10.1177/03000605251375552 (PMC12536115; doi:10.1177/03000605251375552)
Supplement: sj-pdf-2-imr-10.1177_03000605251375552 - Supplemental material for Utility of admission biomarkers in predicting severe outcomes and triage in acute febrile illness: A cohort study [file sj-pdf-2-imr-10.1177_03000605251375552.pdf]

Supplementary Material 2: Comparison of initial admission biomarkers in mild MOD group versus moderate-severe MOD group.

**Biochemical Parameters**	**Mild organ dysfunction SOFA score 0-7 Mean±SD/Median (IQR) (n=35)**	**Moderate to severe organ dysfunction SOFA score 8-24 Mean±SD/Median (IQR) (n=60)**	**P-value**
AST (IU/L)	54 (34.5- 116)	155 (81-236.25)	**0.001***
WBC ($10^3/\mu L$)	7.4 (5.2-12.1)	11.2 (7.15-15.4)	**0.023***
CRP (mg/dL)	129 (27.17-199.95)	215 (146.5-327)	**<0.001***
PCT (ng/mL)	1.84 (0.79-8.94)	12.38 (3.54-46.7)	**<0.001***
Urea (mg/dL)	41(23-64)	97 (62.5-150)	**<0.001***
Sodium (mEq/L)	133.26±5.58	131.77±7.32	0.301**
Potassium (mEq/L)	4.34±0.74	4.48±0.94	0.472**
IL-6 (pg/mL)	64.09±36.55 (n=32)	72.03±27.16 (n=55)	0.251**
NGAL (ng/mL)	155.99±36.22 (n=31)	168.48±35.72 (n=54)	0.126**
iNOS (U/L)	53.79±16.84 (n=32)	56.10±11.97 (n=54)	0.462**

*Mann-Whitney U test; **Unpaired Student's t-test; IL-6: Interleukin-6; NGAL: Neutrophil Gelatinase-Associated Lipocalin; iNOS: Inducible Nitric Oxide Synthase; WBC: White Blood Cells; AST: Aspartate Aminotransferase; CRP: C-Reactive Protein; PCT: Procalcitonin; MOD: Multi-organ dysfunction; P-value ≤ 0.05 is statistically significant (values marked in bold).
