# Supplementary material for: Utility of admission biomarkers in predicting severe outcomes and triage in acute febrile illness: A cohort study
Source: J Int Med Res. 2025 Oct 15;53(10):03000605251375552. doi: 10.1177/03000605251375552 (PMC12536115; doi:10.1177/03000605251375552)
Supplement: sj-pdf-3-imr-10.1177_03000605251375552 - Supplemental material for Utility of admission biomarkers in predicting severe outcomes and triage in acute febrile illness: A cohort study [file sj-pdf-3-imr-10.1177_03000605251375552.pdf]

Supplementary Material 3: Difference in the biomarkers (after admission in Emergency Department) in the AFI patients between non- IMV and the IMV groups.

**Biochemical Parameter**	**No IMV in 48 hours after admission (n=72)**	**IMV required in 48 hours after admission (n=23)**	**P-value**
Bilirubin (mg/dL)	2.55 (0.78-7.41)	4.42 (2.46-6.55)	0.168*
AST (U/L)	89 (44-161)	167 (81-297)	**0.016***
WBC (10^3/µL)	8.05 (6.0-13.07)	11.8 (9.0-15.5)	**0.019***
Platelet (10^3/µL)	40.5(19.5-98.0)	52.0 (18- 136)	0.979*
CRP (mg/dL)	184 (106.2-251.5)	212 (113.15-344)	0.151*
PCT (ng/mL)	4.83(1.18-29.42)	10.4 (2.27-44.94)	0.1908
Urea (mg/dL)	62 (36.25-112.75)	101 (67-156)	**0.006***
Creatinine (mg/dL)	1.78 (1.11-3.42)	3.69 (1.92- 5.08)	**0.004***
Sodium (mEq/L)	131.86±6.39	133.74±7.84	0.247**
Potassium (mEq/L)	4.29±0.77	4.87±1.03	**0.005****
IL-6 (pg/mL)	63.67±31.92 (n=66)	86.18±20.27 (n=21)	**0.003****
NGAL (ng/mL)	160.01±33.50 (n=65)	176.64±42.30 (n=20)	0.072**
iNOS (U/L)	55.54±15.27 (n=66)	54.27±8.27 (n = 20)	0.724**

*Mann-Whitney U test; **Unpaired Student's t-test; IL-6: Interleukin-6; NGAL: Neutrophil Gelatinase-Associated Lipocalin; iNOS: Inducible Nitric Oxide Synthase; WBC: White Blood Cells; AST: Aspartate Aminotransferase; CRP: C-Reactive Protein; PCT: Procalcitonin; P-value ≤ 0.05 is statistically significant (values marked in bold).
